# Association of disease severity and genetic variation during primary Respiratory Syncytial Virus infections

**DOI:** 10.1186/s12920-024-01930-7

**Published:** 2024-06-19

**Authors:** William Bender, Yun Zhang, Anthony Corbett, Chinyi Chu, Alexander Grier, Lu Wang, Xing Qiu, Matthew N. McCall, David J. Topham, Edward E. Walsh, Thomas J. Mariani, Richard Scheuermann, Mary T. Caserta, Christopher S. Anderson

**Affiliations:** 1 Division of Infectious Disease, Department of Medicine, School of Medicine and Dentistry, University of Rochester, University of Rochester Medical Center, Rochester, NY USA; 2 J. Craig Venter Institute, San Diego, CA USA; 3 Department of Biostatistics and Computational Biology, University of Rochester Medical Center, Rochester, NY USA; 4 Division of Neonatology, Department of Pediatrics, University of Rochester Medical Center, Rochester, NY USA; 5 Department of Microbiology and Immunology, University of Rochester Medical Center, Rochester, NY USA; 6 Division of Infectious Diseases, Department of Pediatrics, University of Rochester Medical Center, Rochester, NY USA

**Keywords:** RSV, Whole-genome, Genetic variation, Severe disease, Respiratory infection

## Abstract

**Background:**

Respiratory Syncytial Virus (RSV) disease in young children ranges from mild cold symptoms to severe symptoms that require hospitalization and sometimes result in death. Studies have shown a statistical association between RSV subtype or phylogenic lineage and RSV disease severity, although these results have been inconsistent. Associations between variation within RSV gene coding regions or residues and RSV disease severity has been largely unexplored.

**Methods:**

Nasal swabs from children (< 8 months-old) infected with RSV in Rochester, NY between 1977–1998 clinically presenting with either mild or severe disease during their first cold-season were used. Whole-genome RSV sequences were obtained using overlapping PCR and next-generation sequencing. Both whole-genome phylogenetic and non-phylogenetic statistical approaches were performed to associate RSV genotype with disease severity.

**Results:**

The RSVB subtype was statistically associated with disease severity. A significant association between phylogenetic clustering of mild/severe traits and disease severity was also found. GA1 clade sequences were associated with severe disease while GB1 was significantly associated with mild disease. Both G and M2-2 gene variation was significantly associated with disease severity. We identified 16 residues in the G gene and 3 in the M2-2 RSV gene associated with disease severity.

**Conclusion:**

These results suggest that phylogenetic lineage and the genetic variability in G or M2-2 genes of RSV may contribute to disease severity in young children undergoing their first infection.

## Introduction

RSV infection most often presents clinically as a mild respiratory disease with symptoms of rhinitis and cough. For some individuals, especially young children during their first cold season, the virus presents as severe disease with severe fever, cough, and wheeze leading to significant morbidity and sometimes death [1–4]. Studies have shown an association between severe RSV disease and increased incidence of asthma and allergic disease in young children [3, 5]. Understanding the risk factors associated with RSV disease severity in children has been a continuous subject of study in the RSV field.

RSV viruses can be grouped into two subtypes (RSVA and RSVB). Early studies found RSVA had a greater association with severe disease than RSVB [6–11] while other studies showed a worse disease outcome with RSVB [12, 13], or no difference [14–18], or differences specific to the symptom [13].

RSV can be further subclassified into phylogenetic clade genotypes [19, 20] using the second hypervariable region of the RSV G protein [21, 22]. Association with RSV genotypes has also been variable between studies with some showing clades GA3 being associated, but no other A or B clades [16], some showing NA1 genotypes as causing more frequent lower respiratory tract infections [23]. With the emergence of a new genotype (ON1) for RSVA due to a duplication in the G protein studies have shown variable results with some showing increased severity in individuals infected with ON1 compared to NA1 [24] while others showing milder disease with ON1 [15, 25] and others showing no association with genotype [26]. Still others showed associations with disease severity in some sub-clades of ON1, but not others [27]. Further studies have shown association with specific mutations in the RSV G gene [28, 29].

Several positively selected sites have been identified on the RSV suggesting an adaption to external pressure [30]. Twelve positively selective sites in the RSV B protein were identified in G protein ectodomain sequences suggesting immune pressure [31]. Infection with RSV A viruses containing 5 specific amino acids substituted in G has been shown to less often presented with wheezing [29]. Moreover, mutations in the central conserved domain of G alter the host immune response and decrease severity [32–35]. Additionally, mutations in the F protein result in changes in the host immune response to RSV infection and increased RSV-induced disease severity in mice [12]. Significant genetic variation occurs in other RSV genes besides G including NS2 and M2-1 and M2-2 [36]. Moreover, M2-2 has been found to be under positive selection [22].

Whether variation in RSV genes is associated with disease severity has been largely unexplored. Furthermore, genetic variation that does not affect phylogeny may still increase the likelihood of disease severity but has not been assessed [37].

## Methods

### Sample selection

Subject identifiers for cryogenically frozen nasal swab samples from RSV positive individuals that were collected routinely from patients in both inpatient and outpatient settings in the Rochester, New York area from 1977–1998 and used for a variety of research studies. Subjects were limited to those with phenotypic and clinical information, including age and notes on clinical presentation. Samples were randomly selected for subjects that were in their first RSV season and with no documented previous RSV infection resulting in subjects between 0 and 8 months old of age.

### Mild and severe disease grouping

Severe disease cases was defined as those admitted to the hospital for RSV infections (inpatient) while mild cases consisted of RSV positive cases not requiring hospitalization (outpatient). Using archived medical records, subjects were first grouped into severe and mild based on impatient or outpatient status. Second, individual records were clinically-evaluated and subjects were removed whose symptoms did not match the mild or severe phenotype or did not contain adequate notes to determine severity level.

### Whole-genome RSV sequencing

Whole-genome RSV sequences were generated using overlapping PCR amplicons spanning the RSV genome. The amplicons were pooled by sample, barcoded, and sequenced using the Ion Torrent PGM Next Generation Sequencing platform (355.5 × sequencing depth). The RSV reference (clc_ref_assemble_long v. 3.22.55705) was used for assembly. The consensus sequences of the internal PCR primer hybridization sites were manually verified using reads from amplicons that spanned across the sites. The final dataset contained 160 whole-genome sequences.

### Phylogenetic tree

Whole-genome RSV sequences were translated in silico into amino acids sequences and aligned with ClustalOmega using the MSA package [38] for R Statistical Software version 4.2.2. BEAUti v1.10.4 [39] was used to create an XML document from our aligned AA sequences. Tip dates were set to the sample collection year, a HKY substitution model was used and the Site Heterogeneity Model was set to Gamma model with gamma number of 4. An uncorrelated relaxed clock was utilized. The Tree Prior was set to Coalescent: Bayesian SkyGrid, the number of parameters equal to the number of sequences, with a time at last transition point set to 1.0. The length of MCMC chain was set to 10,000,000 with echo state and log parameters set to 1000. XML files were input into BEAST v1.10.4 [39] and 5 independent runs were performed and combined with Logcombiner and highest credibility tree was determined with Treeannotator. The phylogenetic tree was visualized using Figtree v1.4.4.

### Phylogeny and trait association

The Bayesian Tip-association Significance testing (BaTS) software [40] was to statistically associate phylogenic topology with disease severity (mild/severe). The BaTS algorithm was used to apply three statistical methods to test the association between phylogeny and a trait: parsimony score, association index, and maximum exclusive single-state clade size [40].

### Genotype assignment

Sequences were assigned to RSV genotype clades using genotype reference sequences [21]. Statistical association of RSV genotype and disease severity (mild/severe) was performed using Pearson’s Chi-Square test and permutation test (R v4.2.2).

### Association of viral-gene coding-sequence variation and disease severity using ssTA

Here we introduce a statistical approach based on an immunological shape space [41] called the Shape Space Trait Association (ssTA) algorithm (https://github.com/wbender1/ssTA) ssTA uses viral-gene coding-sequence as input and places each sequence in a genetic distance space in which the distance between any two sequences in the space represents the number of amino acid differences between the coding sequences [42, 43]. Categorical traits, such mild/severe disease, can then be associated with the sequences distribution within the genetic distance space using spatial permutation tests [44].

Sequences were translated in silico for each of the 11 protein-coding-regions for each RSV genome. Protein sequences were aligned using the MUSCLE algorithm [45]. Pairwise Hamming distances between all aligned sequences were determine using the “stringdist” package in R version 3.4.4. resulting in a 160 × 160 distance matrix representing all pairwise genetic distances. To determine if the distribution of sequences in the 160-dimensional space was associated with disease severity trait (mild/severe) we used two statistical methods (Adonis2 [46] and Anosim [47] *Vegan* package, R version 3.4.4). For the *adonis2* method, 9999 permutations were performed to determine empirical null. For the *anosim* method, which is less affected by limited degrees of freedom, 9999 permutations were performed to determine empirical null. P values of less than 0.05 after adjustment (BH) was considered significant.

### Association of disease severity and amino acid usage at each residue

The meta-CATS algorithm [48] was used to identify statistically associate residue positions of RSV amino acid sequence with disease severity status (mild/severe). Protein sequences were aligned using MUSCLE. Subtypes (RSVA and RSVB) were tested separately. P values of less than 0.05 were considered significant.

## Results

Subjects were roughly equally split by sex (Table 1; 42% Female and 58% male) and disease severity (mild: 87/160 (54%) and severe: 73/160 (46%)). RSV subtypes were roughly equally represented (RSVA = 58% and RSVB = 42%). The number of samples varied year to year (2—14 samples per year) with an average of 7.27 samples per year over the 21-year time frame.

**Table 1:** Subject Demographics

Characteristic	Total	Mild	Severe	*p*value
Subjects	160	73 (46%)	87 (54%)	-
Age, Yr (SD)	0.29	0.4 (0.17)	0.2 (0.14)	< 0.001
Sex (M,F)	94, 66	42, 31	52, 35	0.77
RSV Strain (A, B)	93, 67	51, 22	42, 45	0.009

**Fig. 1:**
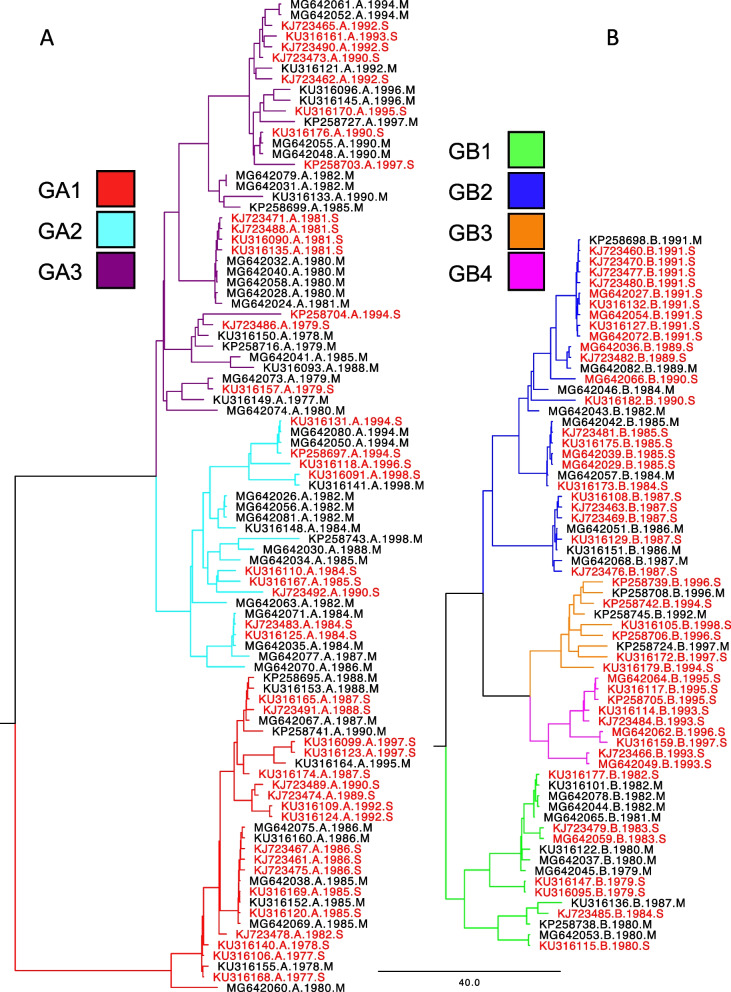
Genomic Variation of the RSV Genome. Sequence genotyping resulted in 7 genotypes (3 RSVA, 4 RSVB). Subtype A contained 93 total sequences falling into 3 different genotypes colored Red(GA1), Cyan(GA2), and Purple(GA3). Subtype B contained 67 total sequences and the 4 different genotypes are colored Green(GB1), Blue(GB2), Orange(GB3), and Pink(GB4). Each sequence name contains metadata representing the subtype, year, and severity (name.subtype.year.severity). Severity is colored Black (Mild) and Red (Severe)

RSV strains separated into two distinct linages corresponding to the RSV A and B subtypes (Fig. 1). We found a significant association with mild/severe and subtype (Chi-square p-value 0.002) with RSVB more likely of being a severe case compared to RSVA. RSVA contained 93 total sequences and was found to be represented by 3 different genotypes: GA1 (*n* = 30), GA2 (*n* = 24), GA3 (*n* = 39), Table 2. RSVB contained 67 total sequences and was represented by 4 different genotypes: GB1 (*n* = 17), GB2 (*n* = 32), GB3 (*n* = 9), GB4 (*n* = 9). GA1 was significantly associated with severe disease (*p*-value = 0.047) while GB1 was significantly associated with mild disease (*p*-value = 0.008). The GB4 genotype was exclusively seen in severe cases.

**Table 2:** Association of Genotype and Severity

	Mild (#seqs)	Severe (#seqs)	*p*-value
GA1	12	18	0.047
GA2	15	9	0.381
GA3	24	15	0.269
GB1	10	7	0.008
GB2	9	23	0.432
GB3	3	6	0.972
GB4	0	9	-

Phylogeny-trait association demonstrated significant differences between the distribution of mild/disease traits and tree topology (Table 3). The association index, which tests the association between traits (mild/severe) and phenotypic clustering, was significant (*p*-value = 0.023) between phylogenetic cluster and disease severity. Additionally, the parsimony score, which determines the number of state changes required to explain the observed trait distribution in the phylogenetic tree, showed a significant association (*p*-value = 0.012) between disease severity and phylogeny. Lastly, the maximum exclusive single-state clade size, which is expected to be larger when tips all share the same trait, were significantly associated for the severe trait (*p*-value = 0.027), but not mild (*p*-value = 0.503).

**Table 3:** Association of Phylogeny and Severity

Statistic	Observed	Lower	Upper	Null mean	Lower	Upper	Significance
	mean	95% CI	95% CU		95% CI	95% CI	
AI	7.025	6.305	7.752	8.862	7.387	10.371	0.023
PS	45.935	44.000	47.000	53.798	48.308	58.778	0.012
MC (Severe)	9.000	9.000	9.000	4.809	3.153	7.233	0.027
MC (Mild)	4.022	4.000	4.000	3.896	2.632	6.000	0.503

**Fig. 2:**
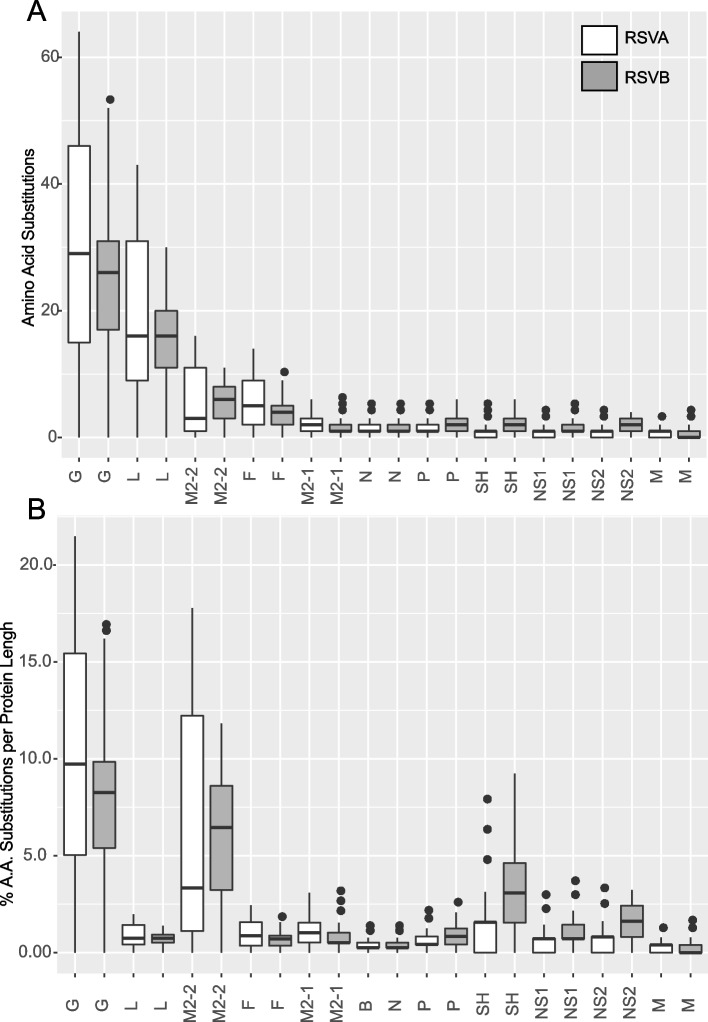
Comparison of Amino Acid Variation in Across RSV Proteins. The number of amino acid substitution between each gene coding region of the whole-genome RSV sequence was calculated. **A** Boxplot of the number of amino acid substitutions for each gene within subtype for each RSV gene coding region. **B** Boxplot of the percentage of number of amino acid substitutions divided by the amino acid length of the coding sequence within subtype for each RSV gene coding region

Of the 11 RSV protein coding regions, the G protein, for both subtypes, showed the maximum number of total amino acid substitutions (RSVA G protein = 64, RSVB G protein = 53; Fig. 2A) as well as the greatest percent change per amino acid length of any protein (RSVA G protein = 21%, RSVB G protein = 17%; Fig. 2B). The M2-2 protein was also one of the most variable proteins both in the number of total amino acids (RSVA M2-2 protein = 16, RSVB M2-2 protein = 11) and percent change per protein length (RSVA M2-2 protein = 18%, RSVB M2-2 protein = 12%). The L protein showed many substitutions for both subtypes (RSVA L protein = 43, RSVB L protein = 30) although the per amino acid change was moderate (RSVA L protein = 2%, RSVB L protein = 1%). Alternatively, the SH protein and F protein showed lower numbers of amino acid substitutions (RSVA SH protein = 5, RSVB SH protein = 6; RSVA F protein = 14, RSVB F protein = 10), but SH showed a moderate change per amino acid compared to other proteins (RSVA SH protein = 8%, RSVB SH protein = 9%) and the F protein showed a minimal number of substitutions per protein length (RSVA F protein = 2%, RSVB F protein = 2%). All other proteins showed both minimal substitutions (3—6) and percent changes (1 – 4%).

**Fig. 3:**
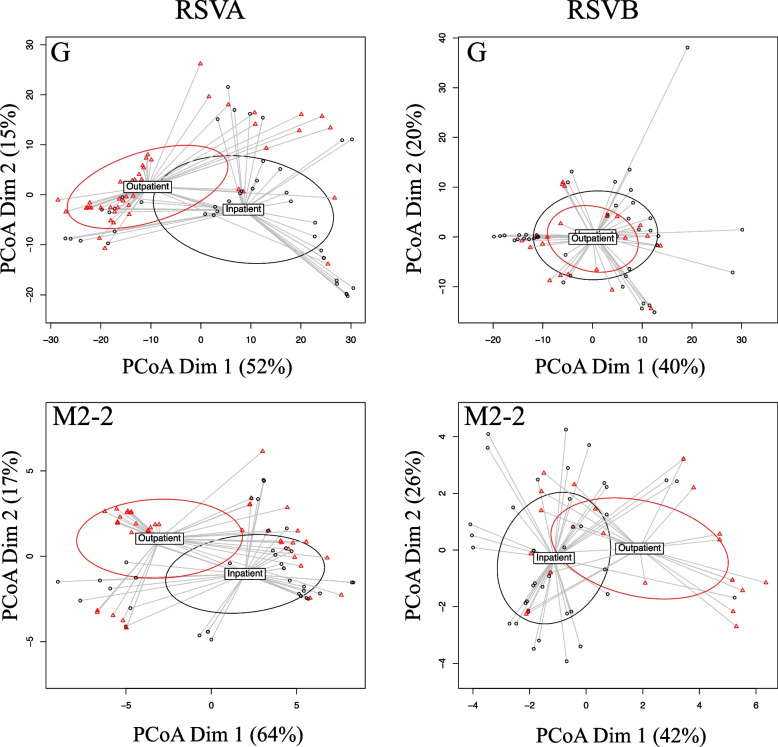
Amino Acid Variability Among RSV G and M2-2 Proteins. Protein sequences for G and M2-2 proteins from RSVA and RSVB subtypes were aligned separately. The number of amino acid substitutions were calculated between all strains and Principal Coordinate Analysis was performed to demonstrate amino acid variability in reduced dimensional space. Ellipses are centered on centroids with 1 standard deviation. Points are colored by disease severity status; red = mild, black = severe. When points contain multiple sequences and from patients of both disease types, points are colored by the more numerous disease type

The G protein for RSVA was significantly associated with disease severity for both statistical tests (Table 4; Fig. 3). The M2-2 protein of RSVA and RSVB were significantly associated with disease severity for both statistical tests. The NS2 protein was also significantly associated with disease severity in the RSVB subtype, but only for one statistical test.

**Table 4:** Association of Protein Variation and Severity

Protein	Subtype	Anosim Rval	Anosim pval	Anosim pval.adj	Adonis2 Fval	Adonis2 pval	Adonis2 pval.adj
G	A	0.122	0.001	0.011	9.438	0.001	0.011
G	B	-0.140	0.999	0.999	-4.832	1.000	1.000
L	A	0.026	0.068	0.166	2.916	0.071	0.137
L	B	-0.005	0.515	0.629	2.083	0.081	0.137
M2-2	A	0.119	0.001	0.011	7.251	0.004	0.022
M2-2	B	0.105	0.007	0.051	5.078	0.002	0.015
F	A	0.038	0.040	0.126	2.602	0.093	0.138
F	B	0.085	0.068	0.166	3.573	0.023	0.072
M2-1	A	0.014	0.153	0.259	2.843	0.073	0.137
M2-1	B	0.040	0.209	0.307	3.178	0.036	0.088
N	A	0.004	0.306	0.396	2.398	0.100	0.138
N	B	0.101	0.024	0.114	2.179	0.095	0.138
P	A	0.011	0.197	0.307	1.129	0.337	0.412
P	B	0.109	0.026	0.114	6.393	0.017	0.072
SH	A	0.020	0.111	0.218	2.676	0.075	0.137
SH	B	-0.079	0.975	0.999	0.327	0.765	0.842
NS1	A	0.041	0.036	0.126	4.224	0.026	0.072
NS1	B	-0.066	0.940	0.999	0.097	0.885	0.927
NS2	A	0.020	0.119	0.218	1.148	0.321	0.412
NS2	B	-0.027	0.695	0.805	7.075	0.001	0.011
M	A	0.021	0.091	0.200	5.191	0.021	0.072
M	B	0.022	0.303	0.396	0.244	0.721	0.835

Association of severity disease and residue position amino acid usage for G and M2-2 sequences was accessed (Table 5). RSVA G-protein had four amino acids associated with severity status, three were found in the Mucin-like-1 domain and one was found in the Heparin-binding-domain (HBD). RSVB G-protein had nine amino acids associated with severity status, three were found in the Mucin-like-1 domain, two were found in the HBD, and four were found in the Mucin-like-2 domain. RSVA M2-2 protein had one amino acid associated with severity status, while the RSVB M2-2 protein had two amino acids associated.

**Table 5:** Association of Residue Variation and Severity

Subtype	Protein	Position	Chisquare Value	*P*-value	MC *P*-value	Degree Freedom	Severe Residue Diversity	Mild Residue Diversity
A	G	81	6.830	0.0328	N.S	2	15 N, 35 S	22 N, 20 S
A	G	107	10.405	0.0341	N.S	4	12 I, 2 N, 36 T	18 I, 4 N, 20 T
A	G	110	9.8392	0.0432	N.S	4	11 L, 39 Q	14 L, 4 P, 24 Q
A	G	197	6.1812	0.0454	N.S	2	39 K, 11 R	24 K, 18 R
B	G	77	6.1049	0.0472	0.0408	2	22 Q	36 Q, 9 S
B	G	137	7.3658	0.0251	0.0305	2	10 I, 12 T	7 I, 38 T
B	G	150	7.3658	0.0251	0.0305	2	12 P, 10 S	38 P, 7 S
B	G	208	7.3658	0.0251	0.0305	2	10 I, 12 T	7 I, 38 T
B	G	222	10.1967	0.0372	N.S	4	10 M, 12 T	7 A, 7 M, 31 T
B	G	246	7.4062	0.0246	N.S	2	22 T	45 T
B	G	285	7.1572	0.0279	0.0252	2	15 S, 7 Y	42 S, 3 Y
B	G	291	13.6980	0.0331	N.S	6	12 P, 10 S	2 F, 5 L, 33 P, 5 S
B	G	300	9.7366	0.04510	N.S	4	10 I, 12 T	7 I, 38 T
A	M2-2	71	6.4908	0.0389	N.S	2	38 N, 11 S	23 N, 18 S
B	M2-2	31	7.7613	0.0206	0.0238	2	7 I, 15 V	29 I, 14 V
B	M2-2	43	6.3626	0.0415	N.S	2	21 N, 1 S	34 N, 9 S

## Discussion

Severe RSV disease is multifactual and the contribution of the virus genetics is still debated. Here we sought to provide evidence RSV-associated severe respiratory disease in young children (0–8 months old) experiencing their primary infection is associated with virus genotype. In the study, we assessed genomic variation of RSV viruses that circulated in Rochester, New York from 1977 – 1998. Our findings agree with others that the RSV genotype changes over time and multiple genotypes circle each year demonstrating that local regions are exposed to a multitude of genetically unique RSV variants each year [19, 49–51].

We compared RSV sequence variation and disease severity using both phylogenetic and non-phylogenetic approaches. Phylogenetic approaches demonstrated that specific RSVA and RSVB genotypes (GA1 and GB1) were associated with disease severity and GB4 was exclusively seen in severe cases. This is in contrast with other studies showing that GA3 was more associated with increased disease severity [16], but given the difference in genotypes and years in which sequences were sampled it is difficult to make direct comparisons. For instance, in our study both GB3 and GB4 had less than ten sequences, making comparisons between mild and severe cases difficult to interpret. Interestingly, phylogenetic tree topography, including monophyletic clades, were associated with severe disease suggesting that disease severity may be tied to RSV evolution as previously suggested [16, 52, 53].

Using a non-phylogenetic approach, we found that variation in specific RSV genes were associated with disease severity. Specifically, the G protein from RSVA was associated with disease severity, but not G from RSVB. It is perhaps not surprising given the G variation was associated with disease severity given the affect the G protein has on attachment to the host [32, 33], immune cell migration [32], host response [34, 54, 55], and antigenic differences [56]. RSVA also showed greater variation over the time period compared to RSVB which may have contributed.

We found that M2-2 protein variation was associated with disease severity for both RSVA and RSVB. M2-2 has been shown to be involved in the regulation of viral RNA transcription and replication balance. RSV viruses with a deletion of M2-2 show decreased virion production and increased protein creation [57]. For that reason, a current RSV vaccine candidate uses an attenuated-virus with an M2-2 gene deletion [57–59]. This work suggests that variation of the M2-2 gene may affect the transcription/replication regulation leading to differences in host disease severity.

A significant association of RSVB NS2 variation and disease severity was found, but only for the adonis2 test, possibly because NS2 gene showed limited variability and the Anosim test is affected when there are limited degrees of freedom. The inconsistency between the two statistical tests make the NS2 results inconclusive. Given the role NS2 plays in immunomodulation [60, 61], additional studies will be needed to determine if NS2 plays a role in severe disease during primary infection.

The impact specific amino acid substitutions RSV have on disease severity is still largely unexplored. State-of-the-art methods were used to associate specific amino acid substitutions in the G or M2-2 proteins with disease severity in young children. Multiple studies have looked at positive selection sites in RSV and have found significantly drifting sites in both G and M2-2. Multiple studies have shown positive selection sites in G [22, 23, 31, 62]. In a cohort of RSV positive infants in Vietnam, M2-2 was found to be under positive selection [22].

Our results suggest that RSV variation in the G or M2-2 G can impact disease severity in in the very young experiencing a primary RSV infection. It is worth noting that age was significantly different between mild and severe subjects and we cannot rule out that our result indicate variants more likely to infect younger children, who are more susceptible to severe disease. Although our studies were not designed to investigate mechanism or causality, they do suggest RSV genes that may play a role in disease severity. Whether these changes that are associate with disease severity arise due to the adaptive pressures, or just random genetic drift, is still unknown and future studies will be needed to confirm mechanistically the effect this variation has on RSV infection and disease. As we approach a new era with newly licensed therapeutics and vaccines, their ability to effect public health may be dependent on their ability to protect against severe variants.

## Data Availability

Sequence data that support the findings of this study were previously deposited to the National Library of Medicine (NCBI) with the primary accession code PRJNA262901.
